# Supplementary material for: Continuously Reinforced Carbon Nanotube Film Sea-Cucumber-like Polyaniline Nanocomposites for Flexible Self-Supporting Energy-Storage Electrode Materials
Source: Nanomaterials (Basel). 2021 Dec 21;12(1):8. doi: 10.3390/nano12010008 (PMC8746542; doi:10.3390/nano12010008)
Supplement: Supplementary file 1 [file nanomaterials-12-00008-s001.zip › nanomaterials-1511513 supplementary.pdf]

nanomaterials

Supplementary Material

Continuously Reinforced Carbon Nanotube Film Sea-Cucumber-Like Polyaniline Nanocomposites for Flexible Self-Supporting Energy-Storage Electrode Materials

Bingjian Li [1], Shi Liu [1], Haicun Yang [1], Xixi Xu [1], Yinjie Zhou [1], Rong Yang [1,2], Yun Zhang [3] and Jinchun Li [1,2,4,*]

[1] School of Materials Science and Engineering, Changzhou University, Changzhou 213164, China; lbj7151@163.com (B.L.); liushi19890101@126.com (S.L.); yhcbobo@cczu.edu.cn (H.Y.); xxx970209@163.com (X.X.); zhouyinjie41@163.com (Y.Z.); cloudyyang@cczu.edu.cn (R.Y.)
[2] Jiangsu Key Laboratory of Environmentally Friendly Polymeric Materials, Changzhou University, Changzhou 213164, China
[3] Changzhou Key Laboratory of Functional Film Materials, Changzhou 213164, China; hannah0422@126.com
[4] National-Local Joint Engineering Research Center of Biomass Refining and High-Quality Utilization, Changzhou 213164, China
* Correspondence: Lijinchun88@163.com

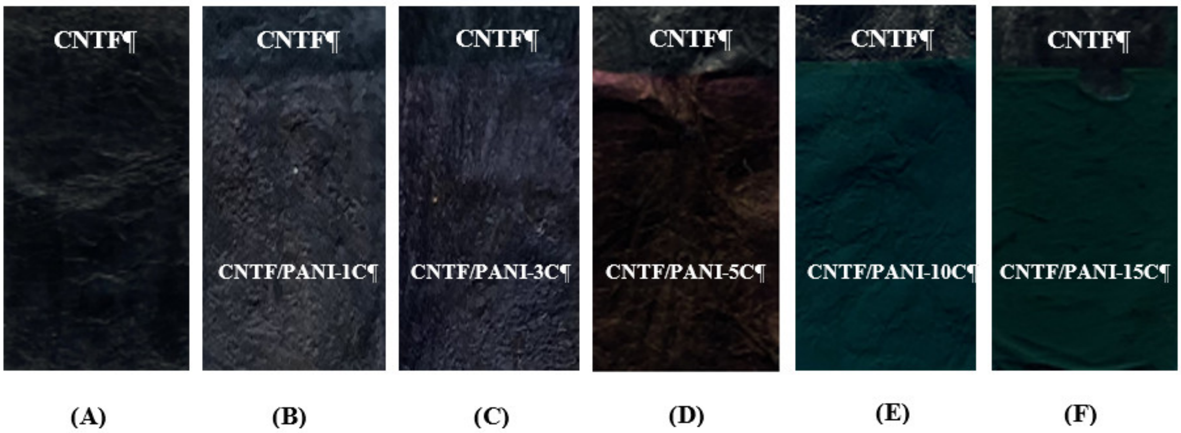

Figure S1. The colors of PANI layer changes during different electrochemical polymerization cycles. Acidified CNTF (**A**), CNTF/PANI-1C nanocomposites (**B**), CNTF/PANI-3C nanocomposites (**C**), CNTF/PANI-5C nanocomposites (**D**), CNTF/PANI-10C nanocomposites (**E**), and CNTF/PANI-15C nanocomposites (**F**).

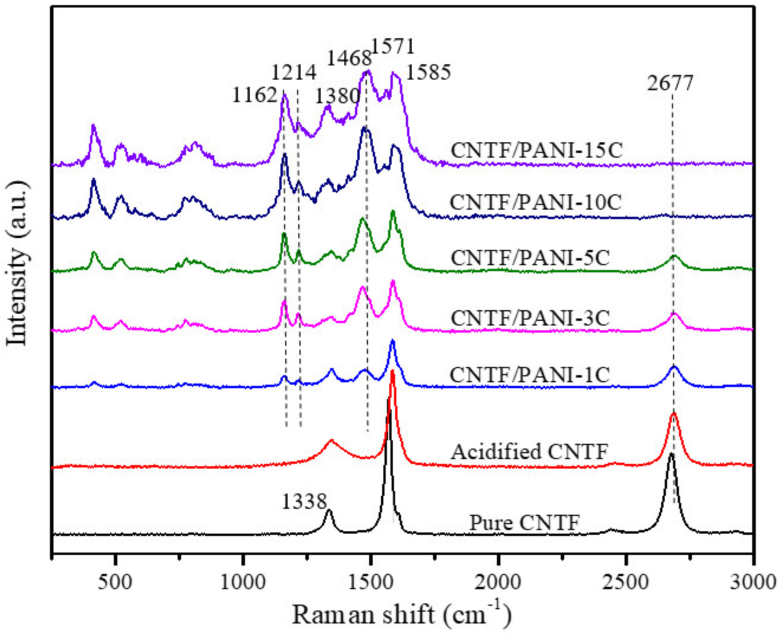

Figure S2. Raman spectra of pure CNTF, acidified CNTF, CNTF/PANI-1C nanocomposites, CNTF/PANI-3C nanocomposites, CNTF/PANI-5C nanocomposites, CNTF/PANI-10C nanocomposites and CNTF/PANI-15C nanocomposites.
